# *Update *on different aspects of HCV variability: focus on NS5B polymerase

**DOI:** 10.1186/1471-2334-14-S5-S1

**Published:** 2014-09-05

**Authors:** Nadia Marascio, Carlo Torti, Maria Carla Liberto, Alfredo Focà

**Affiliations:** 1 Institute of Microbiology, Department of Health Sciences, "Magna Graecia" University of Catanzaro, Catanzaro, Italy; 2 Unit of Infectious Diseases, Department of Medical and Surgical Sciences, "Magna Graecia" University of Catanzaro, Catanzaro, Italy; 3 University Unit of Infectious Diseases, University of Brescia, School of Medicine, Brescia, Italy

## Abstract

The study of hepatitis C virus (HCV) genotypes/subtypes, quasispecies and recombinants obtained by virus genome sequencing are important for epidemiological studies, to trace the source of infection, for development of new direct acting antivirals (DAAs) therapy and for understanding antiviral selection pressures. The HCV NS5B gene encodes a polymerase, which is responsible for virus replication and is a potential target for the development of antiviral agents. Many studies for classification of HCV use a particular segment of the NS5B gene, in addition to other specific regions, and phylogenetic analysis. Actually, some nucleoside/nucleotide analogues and non-nucleoside inhibitors target NS5B protein. This review focuses on HCV variability, phylogenetic analysis and the role of NS5B in the virus-host interactions.

## Introduction

Hepatitis C virus (HCV) is classified as the type member of the genus *Hepacivirus *within the family *Flaviviridae *[1,2]. Nucleotide sequencing allows us to classify HCV into seven genotypes and many subtypes, epidemiologically related to risk factors and geographical areas [3].

HCV genotype determines the duration of antiviral therapy and predicts the response to treatment [4]. In particular viral genotypes influence clinical outcome not only to interferon (IFN)-based therapies but also to IFN free regimens. Classification of HCV into genotypes and subtypes detected by commercial assays is commonly satisfactory to reach a clinical choice. Moreover, the typing of HCV variants, as well as knowledge of the genetic diversity, is important for epidemiological studies such as those to trace the source of infection [5,6].

Phylogenetic and phylogeographical analysis have recently been applied to study the molecular epidemiology of HCV [7-9]. Interestingly, investigation of the spatial and temporal distribution of HCV diversity is critical not only to provide information on the virus origin and history, unknown prior to its identification, but also for understanding mechanism of virus-host interaction, and for preventive strategies [10,3].

Several studies reported heterogeneity in the regions sequenced along the HCV genome, such as 5'-UTR, core, NS5B, HVR-1, E2 and a segment of the NS5A gene associated with interferon sensitivity (ISDR) [11]. Guidelines for classification of genotypes/subtypes, using either the entire genome or the core/E1 and NS5B regions of HCV have been proposed [12,2]. In particular, a segment of the NS5B gene, identifying genomic polymorphisms, has been used [12,2]. HCV NS5B, an RNA dependent RNA polymerase (RdRp), has been studied in various biochemical assays, cell based assays and animal model systems. NS5B variability could be associated with a worse prognosis of the disease, as demonstrated for D310N substitution in HCV 3a infected patients [13].

The aim of the present review is to present the current knowledge of HCV variability for the critical role of NS5B in virus-host interaction.

## NS5B polymerase

### Virological aspects

The viral polymerase NS5B (full-length protein 591 amino acids, *aa*) synthesizes a complementary negative strand RNA using as template genomic positive strand RNA. The catalytic domain, formed by N-terminal 530 aa, exhibits the classical *"fingers", "palm" *and *"thumb" *subdomains typically seen in all RNA dependent RNA polymerases. The active site of NS5B is fully encircled by the fingers and thumb domains, which closely interact. All regular structures, studied until now, reveal a closed conformation, encircled on one side by the fingertips and on the other side by the linker and the so-called *β*-hairpin. Therefore, the active site is fully enclosed and the nucleotide molecules can bind easily with no further rearrangement of the domains [14,15]. As observed in *in vitro *studies, NS5B is able to conduct a template-directed RNA synthesis on its own, requiring only divalent metals (magnesium or manganese) as cofactors. NS5B can also catalyze both *de novo *synthesis from a single-stranded template and primer extension from the subsequent RNA duplex or from a pre-annealed template/primer duplex [16].

HCV RNA replicates in close association with intracellular membranes, so infected cells contain vesicles forming a membranous web (MW) that can be the HCV replication site [17].

Romero-Brey et al. [18], in 2012, carried out an investigation on the 3D morphology and biogenesis of the intracellular membrane structures induced by HCV. The architecture of the membrane alterations induced by HCV reveals unexpected similarities between HCV and the unrelated picorna- and coronaviruses. Therefore, HCV induces, early during an infection, double membrane vesicles (DMVs), which emerge as protrusions of the endoplasmic reticulum (ER). Later on, HCV triggers multi-membrane vesicles (MMVs) that are probably the result of a cellular stress reaction [18]. Consequently, morphology of the MW depends not only on RNA replication, but also on activity of the nonstructural proteins NS3- NS5B, in concert with cellular factors [15]. Indeed, host factors such as lipid kinase phosphatidylinositol 4-kinase III alpha (PI4KIIIα) are essential for RNA replication and interact with NS5B and NS5A proteins. Silencing of PI4KIIIα reduces vesicles formation, suggesting that this enzyme is critically involved in web morphology and consequently in viral replication [19,20].

### Pathogenetical aspects

NS5B binds to several cellular proteins. In particular, it directly interacts with pRb (retinoblastoma - susceptibility protein). The pRb-binding site of NS5B partially overlaps with its polymerase active site and then RdRp activity is blocked. Interaction occurs in both replicon and HCV-infected cells and induces ubiquitination and subsequent degradation of pRb, probably contributing to the oncogenic property of HCV [21].

Liver cirrhosis and hepatocellular carcinoma induced by HCV may involve the interplay of different host cell factors, as well as interaction of these factors with viral RNA and proteins. Upadhyay et al. [22], using an affinity capture system in conjunction with liquid chromatography coupled with tandem mass spectrometry (LC-MS/MS), showed that three viral proteins, NS5B, NS5A and NS3/4A helicase are associated with replicating positive strand RNA genome in hepatoma cells. Moreover, 83 cellular factors such as translation factors, ribosomal proteins, and metabolic enzymes have been found to be associated with HCV genomic RNA. In particular the chaperonin T-complex polypeptide (TCP1), participates in HCV RNA replication and virion production, possibly through its interaction with NS5B. Moreover Caprin 1, a cell-cycle associated phosphoprotein, forms a complex with Ras-GTPase-activating protein-binding protein 1 (G3BP1) which has been shown to interact with both HCV NS5B and the 5' end of the HCV minus-strand RNA indicating that it is part of HCV replication complex [22].

*In vitro *models are important to study viral functional adaptation in human hosts and the natural course of infection [23]. An improvement was achieved after the cloning of a genotype 2a isolates from a Japanese patient with fulminant hepatitis (JFH 1 strain). Intra- and inter-genotypic chimeras, derived from the JFH1 strain, have also been constructed, allowing comparative studies between different genotypes/subtypes [24].

Characterization of JFH1 chimeric genome suggested a role for NS5B during viral assembly [25]. Authors generated a JFH1 chimeric genome, encoding an amantadine-sensitive p7 protein, and identified two point mutations in NS2 and NS5B, which restored the infectivity of defective chimeric genomes. This finding disclosed an association among NS2, NS5B and p7 during virus morphogenesis [25].

### Therapeutical aspects

NS5B represented, over the past decade, a therapeutic target for the inhibition of viral replication. Indeed, the new findings on viral replication, as well as close correlation between viral and host factors, make, even now, the polymerase a good therapeutic target. In addition, two first approved protease inhibitors show a low genetic barrier, inducing rapid onset of drug resistance. Actually, the resistance profiles of DAAs observed in clinical trials are strongly discussed.

Noteworthy alternative drugs are represented by nucleoside/nucleotide analogues, such as sofosbuvir, and non-nucleoside inhibitors, such as BI-207127, both of them directed to NS5B. Currently, sofosbuvir may be associated with ribavirin as therapy for HCV 2 or HCV 3 and with NS5A inhibitors for other genotypes. In HCV genotypes 1 and 4 infection, sofosbuvir/RBV/PEG-IFN may be given for 12 weeks. In persons with HCV 1 infection, IFN intolerant, sofosbuvir/RBV may be given for 24 weeks [26]. BI-207127 is in Phase III development in combination with ribavirin and an NS3 protease inhibitor [27].

Recently a replicon-based reagent suitable for convenient investigation of small molecule-mediated inhibition of HCV genotype 3 NS5A and NS5B has been generated and validated. This replicon chimera represents a useful tool to further study properties/behaviors of polymerase in different HCV genotypes [28].

## HCV variability

### Virological aspects

The positive strand of RNA genome is about 9600 nucleotides long and contains an open reading frame (ORF) encoding polyprotein precursor. The polyprotein is processed into structural and nonstructural proteins by host cell signal peptidases and two viral proteases (NS2 and NS3/4A) [14]. High HCV replication rate, such as 10^12 ^virions per day, and the absence of proofreading activity of NS5B polymerase are the main factors that contribute to mutations in the viral genome. Genome variation is characterized by close genetic diversity among the viruses circulating in an infected host. As a result, HCV exists as a *quasispecies *[29]. HCV polymerase NS5B is responsible of the error mechanism of the gene products. In agreement with its functional role during the viral life cycle, there are few data on diversity within the HCV polymerase.

Recombination, which is an important evolutionary process, is uncommon during the HCV life cycle, although HCV type co-infections are recognized and recombination among genotypes would be expected [30]. Viral recombination has been demonstrated during HCV infection [31] and recently re-examined [32] in view of the low probability to detect recombinant strains and considering the potential underestimation of their incidence.

Viral variants have an impact on immunologic escape and/or antiviral drug resistance and the latter is particularly important in the DAAs (direct acting antivirals) era, when drugs act directly on the specific viral protein. Di Maio et. al [33] analyzed the NS5B polymerase variability in circulating HCV genotypes/subtypes and its impact on the genetic barrier for the development of resistance to clinically relevant nucleoside inhibitors (NIs)/non-nucleoside inhibitors (NNIs).

### Pathogenetical aspects

Blackard et al. [34] detected NS5B intrapatient variability by a greater median genetic distance in the HIV/HCV-coinfected women than in the HCV-monoinfected women. Since HCV RNA levels appear to be significantly increased during HIV/HCV coinfection and HIV coinfection is associated with a reduced drug therapy response, authors conclude that HIV can affect NS5B variability. This is intriguing as this suggests the host immune system, compromised by HIV, can influence genetic diversity of HCV. Functional characterization of unique NS5B sequences, identified *in vivo*, could demonstrate how NS5B variability influences viral replication fitness [34].

### Therapeutical aspects

The effect of NS5B heterogeneity on the activity of nucleoside and non-nucleoside HCV polymerase inhibitors was investigated in clinical isolates obtained from serum samples of treatment-naive patients. It has been shown that non- nucleos(t)ide resistance mutations are present in the viral population at a low frequency in their *quasispecies *and these variants can be promptly selected upon drug pressure [35]. In particular, three major NNI resistant variants (414T, 419S, and 422K) were associated with a different genetic barrier score development among the six HCV genotypes. HCV NS5B sequencing could help to provide efficacy of NNI-containing regimens [33]. Blackard et al. [34] assessed serum NS5B variability in a subset of genotype 1-infected women, which included both HIV/HCV coinfected and HCV monoinfected patients, demonstrated a link between NS5B viral diversity and viral load that could influence therapy outcomes. Among the partial NS5B consensus sequences generated, 21 of 114 (18.4%) amino acid positions were variable. Moreover, recombination could implement antiviral resistances because two different mutations could be brought together a viral recombination event [32].

## Phylogenetic analysis

### Epidemiological aspects

The epidemiology of HCV infection is rapidly changing and HCV prevalence rates between and within countries is characterized by local variability. Risk factors and genotype distribution also have significant geographic and temporal differences. Sequence variability analysis is important for molecular epidemiological studies and for understanding the endemic or epidemic origin of HCV infection. Furthermore, HCV epidemic subtypes show high prevalence, low genetic diversity, and spreading during the 20^th ^century via blood transfusions, injecting drug use and invasive medical procedures. On the contrary, endemic subtypes reveal long persistence in human populations in geographically restricted areas, with low transmission rates and a high genetic diversity as a result [36-38].

Epidemiological changes of HCV infection in Europe, such as in Italy, have been identified. In particular, human migration was responsible for early genotypes/subtypes distribution and actually remains an important factor in HCV type spreading [39].

In 2012, our group investigated the phylogeny of HCV subtype 4d isolated from Italian patients in Calabria (Southern Italy) by analyzing a fragment of the NS5B viral genomic region. A Bayesian coalescent-based framework was used to assess origin and spread of the HCV 4d in this area. Phylogenetic analysis indicated that genotype 4 circulated in Calabria region with a predominance of subtype 4d. Moreover, the Italian 4d isolates shared a common ancestor with reference 4d isolates whose origin was traced back to 1940s (1913-1968). This finding suggests that spread of genotype 4d probably began during the Second World War by immigration from Africa [8].

To assign an accurate genotype/subtype and to identify a new type, sequences of NS5B genomic region and phylogenetic analysis that reconstructed HCV spreading are required [40,5].

In 2002, Richter reported that the NS5B region is the "gold standard" for accurate subtyping [41]. In the same year, Kalinina et al. isolated the first recombinant HCV strain. These authors identified an inter-genotype recombinant between a subtype 2k and a subtype 1b. In this study, the recombinant forms (RFs) were detected performing phylogenetic analysis on 5' UTR-core and NS5B regions [31].

Du et al. [42] detected firstly HCV 3a/1b recombinant through discrepancy of core/E2 and NS5B fragments sequences. HCV recombination and dual infection are often associated with high-risk multiple exposure behaviors. Detection of 3a/1b dual infection not only supports the existence of HCV 3a/1b recombinant, but also indicates a possibility of on-going generation of HCV inter-subtype recombinants [42].

### Pathogenetical aspects

Genetic characterization of animal homologs of human viruses gives information on HCV natural history, evolution and preventive and therapeutic interventions. Kapoor et al. [10], reported the significant finding of HCV homologous *non-primate virus*. Phylogenetic analysis of conserved regions in NS3 and NS5B genes of rodent virus demonstrated homologous sequences with human viruses. The homology allows to hypothesize a zoonotic source of infection and suggests development of new animal models useful to investigate HCV pathogenesis and treatment. In particular, comparative genetic analysis and functional characterization studies might reveal host specificity determinants and different tissue tropism [10]. Extrahepatic replication of HCV in peripheral blood mononuclear cells (PBMC) has significant implications for disease progression and treatment outcome. However, few data are available concerning HCV variability in extrahepatic sites. Blackard et al. [43], by phylogenetic analysis of NS5B and E1/HVR1 sequences, evaluated *quasispecies *extrahepatic diversity by assessing NS5B variability in serum and PBMC samples from chronic HCV patients. Moreover, they identified the possible compartmentalization in PBMCs of HCV variants. Adaptive evolution of non-structural genomic regions such as NS5B could contribute to HCV persistence [43].

### Therapeutical aspects

Pickett et al. [44], in 2011, identified a statistically significant evolutionary divergence of subtype 1a HCV isolates into two distinct clades with potentially different phenotypic behaviors that may reflect differences in treatment response. The informative sites distinguishing clade 1 from clade 2 are distributed throughout the genome, but are mainly located in the E1, E2, NS2, NS5A, and NS5B coding regions. In particular, one site within the 5′ UTR and several sites within the NS5B genotyping fragments differ between clades 1 and 2, this information could be helpful to known if clades differ for therapy resistance. Furthermore, several variants are located at codons associated with resistance to protease inhibitors (NS3 Q41) or polymerase inhibitors (NS5B S368) [44].

## Conclusions

Since HCV was isolated, there has been an increase in the number of studies to analyze the virus.

Structural and functional studies of the Hepatitis C virus RNA dependent RNA polymerase have contributed to our understanding of polymerase mechanism, viral RNA replication, and have generated targets for antiviral development. A multidisciplinary approach which could yield significant benefits to society against a disease with enormous worldwide impact is necessary. Major advances and future research questions on NS5B polymerase are reported in Table 1.

**Table 1:** NS5B polymerase advances and future research

Major advances
• NS5B interacts with different host factors for viral replication [14-20] and virus morphogenesis [25]. • NS5B represents a therapeutic target for the inhibition of viral replication [27,35,44]. • NS5B is responsible for high variability of HCV [29]. • NS5B viral genomic region is necessary for phylogenetic and phylodinamic analysis [7-9], but not enough to identify recombinant strains [31,32,42].

**Major research questions**

• Is NS5B variability associated with prognosis of the disease in HCV infected patients? [13] • Is viral replication fitness influenced in HCV infected and HIV/HCV co-infected patients by NS5B variability? [34] • Can adaptive evolution of NS5B contribute to persistence of HCV in PBMCs? [43]

Nucleotide sequencing with phylogenetic analysis of NS5B genomic region, in addition to core/E1, is recommended for HCV genotyping [2]. Interestingly, the discrepancy of genotyping results between core/E2 and NS5B regions, indicates the presence of a HCV intersubtype recombinant [42].

NS5B region alone may be ineffective to identify HCV recombinants. The heterogeneity of the sequencing regions, in different publications, suggests a necessary standardization of regions analyzed for phylogenetics, phylodynamics and evolutionary studies.

Moreover, viral protein NS5B is a target for therapeutic intervention. The DAA molecules, either alone or in combination with peg-IFN plus RBV, were recently described as showing large antiviral effects. DAA monotherapy results in rapid emergence of resistant strains and DAAs must be used in combinations. The goal of the future studies will be to generate wild type and mutant (intra or inter-genotypes) replicons to investigate emergence and fitness of resistance for NS5B inhibitors.

## List of abbreviations used

HCV: Hepatitis C virus; IFN: interferon; ISDR: interferon sensitivity; DAA s: direct acting antivirals; PBMC: peripheral blood mononuclear cells; HVR1: hypervariable region 1; JFH1: Japanese fulminant hepatitis strain; RFs: recombinant forms; TCP1: chaperonin T-complex polypeptide; G3BP1: Ras-GTPase-activating protein-binding protein 1.

## Competing interests

The authors declare that they have no competing interests.

## Authors' contributions

NM wrote the review article; CT contributed to major review revision; MCL designed and wrote the review article; AF contributed to major review revision.

## Declarations

Publication of this supplement was partly supported by an unrestricted grant provided by Roche. The articles were independently prepared by the authors with no input from Roche. Roche were not involved in selecting the articles for the supplement.

This article has been published as part of *BMC Infectious Diseases *Volume 14 Supplement 5, 2014: Proceedings of the Third Workshop of the HCV Study Group in the Calabria Region: Results from the South Italian Network for Rational Guidelines and International Epidemiology (SINERGIE) Project. The full contents of the supplement are available online at http://www.biomedcentral.com/bmcinfectdis/supplements/14/S5.
